# Transmission electron microscopic observation of body cuticle structures of phoretic and parasitic stages of Parasitaphelenchinae nematodes

**DOI:** 10.1371/journal.pone.0179465

**Published:** 2017-06-16

**Authors:** Taisuke Ekino, Toyoshi Yoshiga, Yuko Takeuchi-Kaneko, Natsumi Kanzaki

**Affiliations:** 1 Laboratory of Nematology, Department of Applied Biological Sciences, Faculty of Agriculture, Saga University, Saga, Japan; 2 The United Graduate School of Agricultural Sciences, Kagoshima University, Kagoshima, Japan; 3 Graduate School of Agriculture, Kyoto University, Sakyo-ku, Kyoto, Japan; 4 Forestry and Forest Products Research Institute, Tsukuba, Ibaraki, Japan; Natural Resources Canada, CANADA

## Abstract

Using transmission electron microscopy, we examined the body cuticle ultrastructures of phoretic and parasitic stages of the parasitaphelenchid nematodes *Bursaphelenchus xylophilus*, *B*. *conicaudatus*, *B*. *luxuriosae*, *B*. *rainulfi*; an unidentified *Bursaphelenchus* species, and an unidentified *Parasitaphelenchus* species. Nematode body cuticles usually consist of three zones, a cortical zone, a median zone, and a basal zone. The phoretic stages of *Bursaphelenchus* spp., isolated from the tracheal systems of longhorn beetles or the elytra of bark beetles, have a thick and radially striated basal zone. In contrast, the parasitic stage of *Parasitaphelenchus* sp., isolated from bark beetle hemocoel, has no radial striations in the basal zone. This difference probably reflects the peculiar ecological characteristics of the phoretic stage. A well-developed basal radially striated zone, composed of very closely linked proteins, is the zone closest to the body wall muscle. Therefore, the striation is necessary for the phoretic species to be able to seek, enter, and depart from host/carrier insects, but is not essential for internal parasites in parasitaphelenchid nematodes. Phylogenetic relationships inferred from near-full-length small subunit ribosomal RNA sequences suggest that the cuticle structures of parasitic species have apomorphic characters, e.g., lack of striation in the basal zone, concurrent with the evolution of insect parasitism from a phoretic life history.

## Introduction

In general, ecdysozoan animals have a more or less sclerotized exoskeleton, which protects their soft tissues from biotic and abiotic factors such as desiccation, osmotic shock, predators, and parasites [1–3]. Nematodes, one of the most divergent animal phyla, also have a cuticular body surface consisting of collagenous protein material [4, 5]. The nematode cuticle is, as for other phyla, one of the most important structures in its body plan. The complex extracellular matrix covering the outermost layer acts as an antagonist to the high internal body pressure and contraction of the longitudinal body muscles [6]. In addition, the body cuticle not only provides shape to its body as also aids mobility and sensing environmental conditions [6]. The nematode cuticle structure is extremely variable, not only among different taxa but also between sexes and across the developmental stages within a species [7–9], reflecting its function in adaption [10]. However, the functional morphology of the nematode body cuticle is not yet sufficiently understood, largely because of its incredible diversity.

In the present study, we examined the body cuticle structure of several parasitaphelenchid (subfamily Parasitaphelenchinae) nematodes, focusing on stages that define their association with their host/carrier insects. The Parasitaphelenchinae nematodes are mostly associated with coleopteran and hymenopteran insects as their phoretic or parasitic hosts. Bark beetles are the most common hosts of this subfamily [11–13], but their associations with ambrosia beetles [14], weevils [15, 16], longhorn beetles [17–19], nitidulids [20], stag beetles [21], and soil-dwelling bees [22, 23] have also been reported across the world. These nematodes have dauer (dormant) or parasitic juvenile stages. For example, dauer juveniles of *Bursaphelenchus* spp. usually stick to the undersurface of the elytra of bark beetles [24, 25] and are capable of entering the tracheal systems of longhorn beetles and weevils [17–19] or the reproductive organs or oviducts, poison sacs, and/or Dufour’s glands of teneral adult digger bees [22, 26, 27]. By contrast, parasitic *Parasitaphelenchus* juveniles enter the hemocoel of insect larvae, pupae, and teneral adults [28–30].

The subfamily contains two lethal plant pathogens, *B*. *xylophilus* and *B*. *cocophilus*, associated with *Monochamus* longhorn beetles as dauer juveniles and with palm weevils as parasitic juveniles [15, 17]. Therefore, the host associations of parasitaphelenchid nematodes are of interest not only for their biological significance but also for disease control.

We investigated the body cuticle ultrastructures of parasitaphelenchid nematodes in relation to their association with insects (as dauer or parasitic juveniles and parasitic adults). We focused on the organs harboring nematodes, dependency on insects (phoresy or parasitism), and nematode developmental stage (juvenile or adult) to understand whether body cuticle structures correspond to these behavioral and/or physiological characteristics. We also compared these structural differences to understand the phylogenetic relationships of nematodes in an evolutionary context.

## Materials and methods

### Insect collection and nematode isolation

Five species of coleopteran insects were collected, dissected to identify parasitaphelenchid nematodes, and examined.

Adult specimens of *Psacothea hilaris* and *Acalolepta luxuriosa* were collected in June 2016 from a mulberry field at the experimental farm of the Kyoto Institute of Technology, Kyoto, Japan (35°01′27″ N, 135°41′02″ E, 45 m above sea level [a.s.l.]) and from *Aralia elata* cultivated at an experimental nursery at the Forestry and Forest Products Research Institute (FFPRI), Ibaraki, Japan (36°00′23″ N, 140°07′33″ E, 24 m a.s.l.), respectively.

Newly emerged *Monochamus alternatus* adults were collected from a dead *Pinus densiflora* log in May 2016. The logs were obtained from the Tama Forest Science Garden, FFPRI, Hachioji, Tokyo, Japan (35°38′44″ N, 139°16′48″ E, 184 m a.s.l), and placed in a wire mesh cage in an experimental field at FFPRI.

To isolate bark beetles (*Dryocoetes uniseriatus* and *Alniphagus costatus*), dead logs obtained from the field were individually enclosed in nylon mesh bags and kept in a wire mesh cage in the FFRPI experimental field. When adult beetles emerged, they were collected using an aspirator. Adult specimens of *D*. *uniseriatus* emerged in May 2016 from a dead *P*. *densiflora* log obtained in May 2016 from the Chiyoda Experimental Nursery of FFPRI, Kasumigaura, Ibaraki, Japan (36°11′11″ N, 140°12′55″ E, 42 m a.s.l). Adult specimens of *A*. *costatus* emerged in July 2016 from several *Alnus serrulatoides* logs obtained in May 2016 from the Sugadaira Montane Research Center, University of Tsukuba, Nagano, Japan (36°31′09″ N, 138°21′00″ E, 1300 m a.s.l).

More than 10 individuals were collected for each of three longhorn beetle species, and more than 100 individuals of both bark beetle species emerged and examined.

A collection permit was not necessary for *A*. *luxuriosa*, *M*. *alternatus*, or *D*. *uniseriatus*, because those were obtained from the experimental field belonging to FFPRI. The collection of *P*. *hilaris* and *A*. *consttus* was permitted by the Kyoto Institute of Technology (Dr. T. Akino) and the University of Tsukuba (Dr. Y. Degawa), respectively.

The insects obtained were identified visually and dissected under a light microscope (S8 Apo, Leica). When nematodes were observed, the organs that harbored these were recorded. Then the developmental stages of nematodes (phoretic [dauer] juvenile, parasitic juvenile, or “parasitic” adult) were observed under a light microscope (Eclipse 80i, Nikon, 200 or 400 X). Phoretic and parasitic stages of nematodes were obtained from one individual each of *P*. *hilaris*, *A*. *luxuriosa* and *A*. *costatus*, two individuals of *M*. *alternatus* and three individuals of *D*. *uniseriatus*. The observed nematode individuals were then saved for molecular identification as described below.

Isolated nematodes were either fixed for transmission electron microscopic (TEM) observations as described below, or transferred to 2.0% malt extract agar previously inoculated with gray mold, *Botrytis cinerea*, to examine their developmental stage (marked third or fourth stage).

### Molecular identification and phylogeny

DNA material was prepared from each nematode species following the methods of Kikuchi *et al*. [31] and Tanaka *et al*. [32]. In brief, a single individual nematode was handpicked from a culture plate or a dissected insect, morphologically observed, and digested in 30 μL nematode digestion buffer at 55°C for 20 min. Nematode lysate served as the polymerase chain reaction (PCR) template. The DNA base sequences of partial ribosomal DNAs, ca. 1.6 kb of nearly-full-length small subunit (SSU) and ca. 750 bp of D2–D3 expansion segments of large subunit, were determined for each isolate following the methods of Kanzaki and Futai [33] and Ye et al. [34] with direct PCR sequencing. The sequences obtained were compared with those in the GenBank database (http://www.genome.jp/dbget-bin/www_bfind?genbank-today).

The molecular phylogenetic relationships of the obtained species were inferred from SSU using Bayesian inference (BI) and maximum likelihood (ML) analyses. The compared sequences were aligned using the MAFFT multiple sequence alignment program [35] (http://mafft.cbrc.jp/alignment/software/), and the base substitution model was determined using the MODELTEST program version 3.7 [36] under the Akaike information criterion (AIC) and the GTR+I+G model was selected for the analysis. The Akaike-based model, log likelihood (lnL), AIC values, proportion of invariable sites, gamma distribution shape parameters, and substitution rates were adopted for both BI and ML analyses. Bayesian analyses were performed using MrBayes 3.2 software [37, 38] by running four chains for 4 × 10^6^ generations. Markov chains were sampled at intervals of 100 generations [39]. Two independent runs were performed and, after confirming the convergence of runs and discarding the first 2 × 10^6^ generations as burn-in, the remaining topologies were used to generate a 50% majority-rule consensus tree. PhyML 3.0 software [40] (http://www.atgc-montpellier.fr/phyml/) was employed for ML analyses. The tree topology was evaluated with 1,000 bootstrap pseudoreplications.

### Observation of body cuticle ultrastructure

Samples for transmission electron microscopy were prepared following the method of Kadoya [41] with some modifications. Phoretic or parasitic stages of nematodes were fixed in 1% glutaraldehyde and 0.6% sucrose in 0.1 M phosphate buffer (pH 7.4) for more than 24 h. The head or tail regions were cut off and left in the same fixative for more than 24 h. After rinsing in the same buffer (six times, 10 min each), the nematodes were post-fixed in 1% osmium tetroxide for 90 min in the same buffer. The fixed nematodes were dehydrated in a graded ethanol series (50%, 70%, 80%, 90%, and three times with 99.5%). Then they were cleaned with propylene oxide (three times, 10 min each) and infiltrated overnight in a mixture of 50% Eponate resin and 50% propylene oxide and an undiluted resin. Finally, the nematodes were embedded in Eponate resin. Their mid-body regions were sectioned with a diamond knife in an ultramicrotome. Sections were collected on formvar-coated copper grids for electron microscopy. The grids were stained with EM stainer (Nisshin EM Co.) for 30 min followed by lead citrate for 5 min. Grid-mounted sections were examined and photographed at 200 kV using a JEOL JEM-2000EX transmission electron microscope. Measurements were taken from body cuticle photographs for zones that were clearly observed. The thickness of each zone and total thickness of the cuticle were measured using the ImageJ program [42] (https://imagej.nih.gov/ij/).

We analyzed differences in cuticle thickness among all species and the thickness of the cuticle zone (%) among *Bursaphelenchus* spp. using the Steel-Dwass test.

## Results

### Nematode isolation

A dome-shaped lip region and less-developed median bulb, are typical morphological characters of insect associated forms of parasitaphelenchids [30, 43–45]. Thus, the nematodes with those characters were chosen as candidate phoretic or parasitic juveniles. Nematodes of *B*. *luxuriosae* obtained from *A*. *luxuriosa* tracheas were identified as “parasitic adults” based on a dome-shaped lip region, well-developed median bulb, and fully developed reproductive system (vulva or spicule) [19, 44, 45]. The parasitic juveniles isolated from the hemocoel of *A*. *costagus* had hooks on both the anterior and posterior ends of the body, suggestive of a typical parasitic juvenile form of the genus *Parasitaphelenchus* [30].

The nematode stages (phoretic juvenile, parasitic juvenile, or parasitic adult), insect vectors, and insect organs that harbored nematodes are summarized in Table 1. The dauer juveniles of *B*. *xylophilus* and *B*. *conicaudatus* and “parasitic” adults of *B*. *luxuriosae*, were isolated from the tracheas of *M*. *alternatus*, *P*. *hilaris* and *A*. *luxuriosa* respectively. The dauer juveniles of *B*. *rainulfi* and the undescribed *Bursaphelenchus* sp. were isolated from the backs of elytra of *D*. *uniceriatus* and *A*. *costatus*, respectively. Parasitic juveniles of the undescribed *Parasitaphelenchus* sp. were isolated from the hemocoel of *A*. *costatus*.

**Table 1:** Species, stages, insect vectors, and insect organs harboring nematodes.

Species	Stage	Insect vector	Insect organ harboring nematodes
***Bursaphelenchus xylophilus***	Fourth stage phoretic juvenile	*Monochamus alternatus* (Cerambycidae)	Trachea
***B*. *conicaudatus***	Fourth stage phoretic juvenile	*Psacothea hilaris* (Cerambycidae)	Trachea
***B*. *luxuriosae***	“Parasitic” adult	*Acalolepta luxuriosa* (Cerambycidae)	Trachea
***B*. *rainulfi***	Third stage phoretic juvenile	*Dryocoetes uniseriatus* (Scolytidae)	Under the elytra
***Bursaphelenchus* sp.**	Third stage phoretic juvenile	*Alniphagus costatus* (Scolytidae)	Under the elytra
***Parasitaphelenchus* sp.**	Third stage parasitic juvenile	*Alniphagus costatus* (Scolytidae)	Hemocoel

The dauer juveniles of *B*. *xylophilus* and *B*. *conicaudatus* were in the fourth stage of dispersion, as previously described [46, 47]. The dauer juveniles of *B*. *rainulfi* and the *Bursaphelenchus* sp. and the parasitic juveniles of the *Parasitaphelenchus* sp. were third stage, which molted to the fourth stage within a week after transfer to the culture plate.

### Molecular identification and phylogeny

The SSU sequences of *B*. *xylophilus*, *B*. *conicaudatus*, *B*. *luxuriosae*, and *B*. *rainulfi* were almost identical to those of other population(s) of the same species deposited in the GenBank database (SSUs showed 100% identity with AY508034, 99% identity with AM397011, 99% identity with AB097864, and 99% identity with AM397017, respectively).

**Fig 1:**
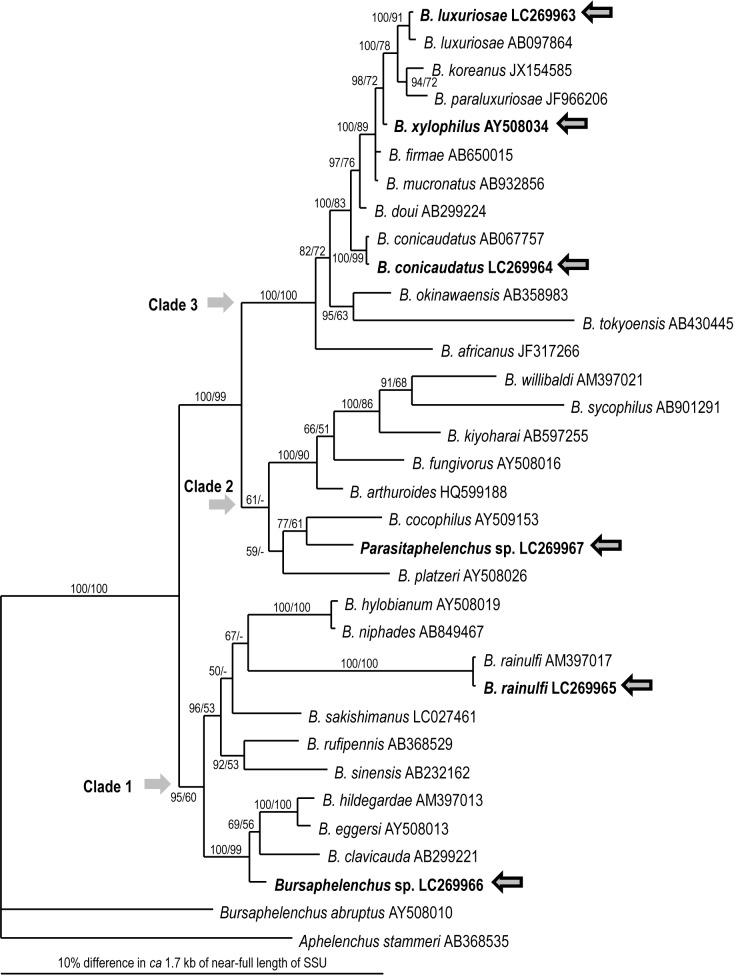
Bayesian inference (BI) tree inferred from near-full-length SSU under the GTR+I+G model. Analytical parameters are as follows: lnL = 6581.5269; freqA = 0.2615; freqC = 0.1826; freqG = 0.2598; freqT = 0.2961; R(a) = 1.0857; R(b) = 3.0396; R(c) = 1.3291; R(d) = 0.4709; R(e) = 6.0774; R(f) = 1; Pinva = 0.6408; Shape = 0.5838. BI posterior probability and bootstrap values obtained from an independent maximum likelihood (ML) analysis exceeding 50% are given on appropriate clades. Values lower than 50% are shown by “–”.

Based on SSU sequences, the *Bursaphelenchus* sp. obtained from the elytra of *A*. *serrulatoides* belongs to clade 1 *sensu* Kanzaki & Giblin-Davis [12] and is closely related to the *B*. *eggersi* and *B*. *eremus* groups [48], e.g., *B*. *eggersi* (AY508013), *B*. *clavicauda* (AB067757), and *B*. *hidegardae* (AM397013) (Fig 1). *Parasitaphelenchus* sp. is a member of clade 2 of the subfamily (≈ *Bursaphelenchus*) *sensu* Kanzaki & Giblin-Davis [12] and is closely related to *B*. *cocophilus* (AY509153) and *B*. *platzeri* (HQ599188) (Fig 1), i.e., the “genus” *Parasitaphelenchus* is phylogenetically a part of a larger “genus” *Bursaphelenchus*.

### Observation of body cuticle ultrastructure

The body cuticle structures of the phoretic and parasitic stages of the parasitaphelenchid species were observed via transmission electron microscopy. The structures of these species can be divided into two types, marked as types 1 and 2.

**Fig 2:**
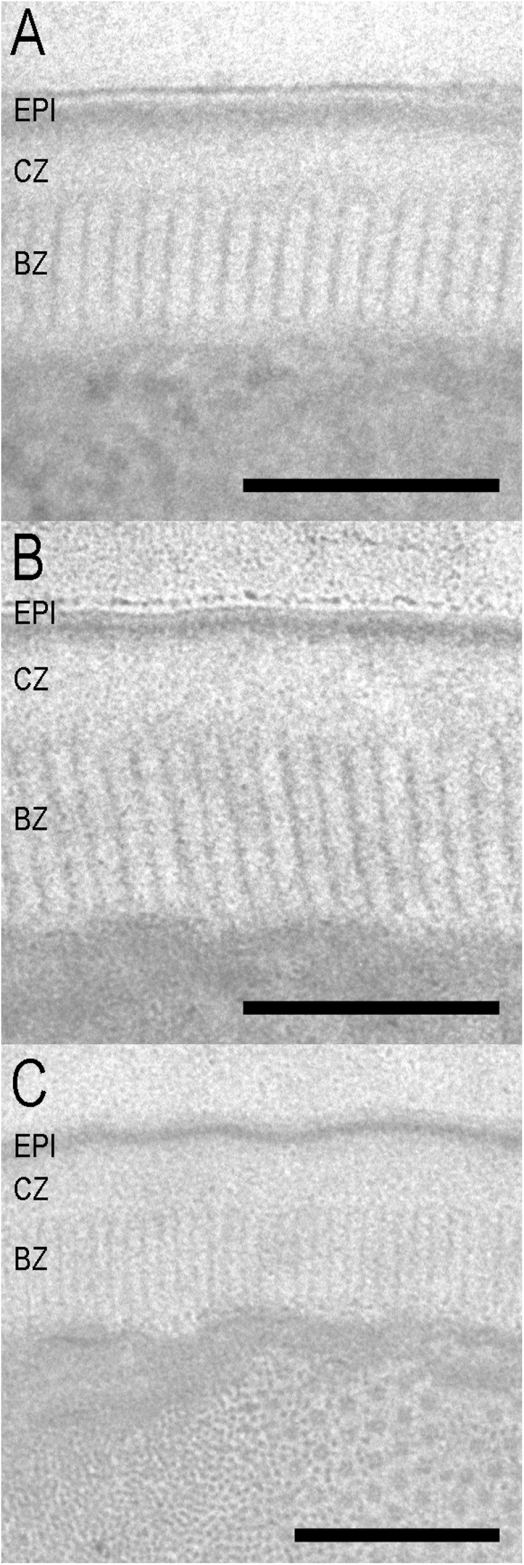
Ultrastructure of the body cuticle of phoretic stages of Parasitaphelenchinae species. A: *B*. *rainulfi*; B: *Bursaphelenchus* sp.; C: *B*. *conicaudatus*; EPI = epicuticle; CZ = cortical zone; BZ = basal zone. Scale bar = 200 nm.

**Fig 3:**
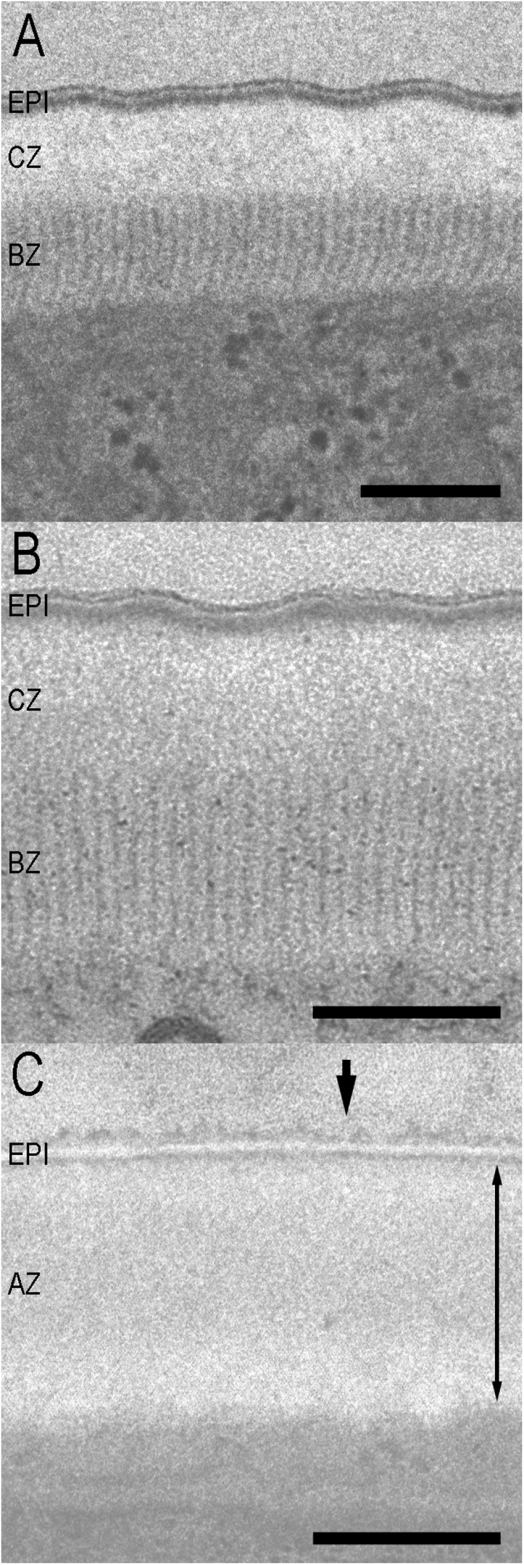
Ultrastructure of the body cuticle of parasitic stages of Parasitaphelenchinae species. A: parasitic male of *B*. *luxuriosae*; B: parasitic female of *B*. *luxuriosae*; C: parasitic juvenile of *Parasitaphelenchus* sp. Arrowhead suggests the faintly stained surface coat. The thickness of the amorphous zone is indicated by the double-headed arrow. EPI = epicuticle; CZ = cortical zone; BZ = basal zone; AZ = amorphous zone. Scale bar = 200 nm.

The structure of type 1 cuticle is the same as the one previously reported for dispersal fourth-stage juveniles of *B*. *xylophilus* [49] (Figs 2, 3A and 3B). Type 1 consists of three parts: a triple-layered epicuticle, a cortical zone, and a basal zone. No median zone was observed in propagative juveniles, adults, or dispersal third-stage juveniles [49]. The epicuticle consists of an electron-dense outermost layer (surface coat) and double inner layers. The cortical zone is an electron-lucent, amorphous zone. The basal zone is radially striated (hereinafter referred to as “striated”) and distinguishable from the cortical layer. All *Bursaphelenchus* spp. dauer juveniles and “parasitic” adult forms have type 1 cuticle structures. The thickness of each zone and the ratio of each zone to the total cuticle are summarized in Table 2. There were no significant differences (P< 0.05) in total cuticle thickness among all compared species nor in the thickness (%) of the cuticle zone among *Bursaphelenchus* spp. The basal striated zone in all *Bursaphelenchus* spp. constitutes the largest proportion (about 50%) of the total cuticle thickness.

**Table 2:** Measurement of cuticle thickness and the ratio of cuticle zone thickness to total cuticle thickness of phoretic and parasitic stage Parasitaphelenchinae species.

Species	n	Thickness of cuticle (nm) ± S.E.	Percentage of the thickness of the cuticle zones in a form, % (range)		
			EPI	CZ	BZ
***Bursaphelenchus*. *xylophilus***	5	238.2 ± 5.2	13.0 (10.9–15.0)	34.0 (28.5–39.1)	53.0 (48.9–56.5)
***B*. *conicaudatus***	5	240.6 ± 10.6	12.3 (11.1–16.7)	34.3 (30.4–37.4)	53.4 (51.0–58.5)
***B*. *luxuriosae* (PM)**	5	272.2 ± 6.9	10.0 (7.8–11.2)	40.0 (26.7–50.8)	50.0 (41.4–53.8)
***B*. *luxuriosae* (PF)**	5	280.2 ± 14.7	11.3 (9.5–12.3)	39.1 (37.1–41.5)	49.6 (46.5–51.9)
***B*. *rainulfi***	5	203.7 ± 5.9	16.7 (14.5–18.9)	30.0 (26.9–34.7)	53.3 (46.4–58.0)
***Bursaphelenchus* sp.**	5	247.4 ± 12.2	12.5 (10.6–16.4)	34.3 (27.1–46.3)	53.2 (43.1–62.4)
***Parasitaphelenchus* sp.**	5	260.3 ± 15.7	8.3 (6.8–9.7)	91.7 (90.3–93.2)	

PM = parasitic male; PF = parasitic female; EPI = epicuticle; CZ = cortical zone; BZ = basal zone. No significant differences were seen in cuticle thickness or the ratio between cuticle zones among species (P<0.05). *Parasitaphelenchus* sp. was excluded from the percentage data comparison because of its structural differences from *Bursaphelenchs* spp.

By contrast, type 2 cuticle (*Parasitaphelenchus* sp.) is characterized by two zones: a triple-layered external epicuticle and an amorphous zone (Fig 3C). The epicuticle consists of an electron-dense outermost layer (surface coat) and double inner layers. The surface coat is faintly stained and unclear. Because the basal zone of type 2 cuticle does not have striation patterns, the cortical, median, and basal zones are not clearly distinct and are composed of an amorphous zone. The thicknesses of an inner double-layered epicuticle and an amorphous zone (cortical, median, and basal zones) and the ratio of each zone to the total cuticle are shown in Table 2.

## Discussion

We obtained primary information on the body cuticle structure of phoretic and parasitic stages of Parasitaphelenchinae species and compared the structures in relation to their biological (mostly behavioral) characteristics and phylogenetic contexts.

The body cuticle structures of dauer juveniles of four *Bursaphelenchus* spp. (*B*. *xylophilus*, *B*. *conicaudatus*, *B*. *rainulfi*, and an undescribed *Bursaphelenchus* sp.) and the parasitic adult form of *B*. *luxuriosae* are similar (type 1), but differ from the parasitic juveniles of an unidentified *Parasitaphelenchus* sp. (type 2).

Kondo and Ishibashi [49] examined the body cuticle structures of dauer juveniles, propagative juveniles, and adult pine wood nematode, *B*. *xylophilus*, and found that the relative thickness of the basal striated zone of dauer juveniles (phoretic stage) is clearly higher (*~*67% of total cuticle) as compared to that of other juveniles and adult stages (*~*27−40%). In the present study, the relative thickness of the basal striated zone in dauer juveniles and parasitic adult forms is around 50% regardless of species (Table 2), thinner than the value reported by Kondo and Ishibashi [49]. We do not have a clear explanation for this difference; however, the relatively thick basal striated zone is common among all insect-dependent forms of *Bursaphelenchus* spp. Generally, the basal striated zone is composed of highly resistant proteins with very close linkage [50], and striation is usually found when nematodes are exposed to hazardous environments (e.g., desiccation, chemicals, variation in osmotic pressure, and mechanical pressure from soil particles) [8, 9, 51, 52]. Therefore, the basal striated zone of these stages is hypothesized to provide resistance to such environments [9, 10]. Furthermore, the basal striated zone is thought to be involved with movement and locomotion, because this zone is closest to the body wall muscle [51, 53–55].

In *B*. *xylophilus*, a well-developed basal striated zone is considered to play an important role in nematode–vector interactions [49, 56]. When phoretic (dispersal stage) juveniles of *B*. *xylophilus* migrate to the body of a vector beetle, the nematodes appear on the surface wall of a vector’s pupal chamber and then climb up the protruded part of the chamber wall to attach to the body of the teneral adult. Then they move into the tracheal system through the spiracles [57].

In the present study, a well-developed basal striated zone is confirmed in *Bursaphelenchus* spp. individuals that were recovered from the tracheal systems of Lamiini longhorn beetles or the backs of elytra of bark beetles. Although the associated organs are different, the mechanism of entry of nematodes into vector is hypothesized to be similar, i.e., they crawl up to teneral adults in the vectors’ pupal chambers. Thus, a well-developed basal striated zone (high mobility), is required for this cascade of activities.

Although the environmental conditions facing nematodes may be different for the associates longhorn and bark beetle, each insect associate is hypothesized to be exposed to strong desiccation. The associates of longhorn beetle have to migrate up tree surfaces until they enter into the wood tissue through a feeding wound or an oviposition mark made by the insect [17, 58–60]. They are exposed to strong desiccation during departure from the vector. On the other hand, bark beetles emerge from dead trees; fly to and invade new host trees; mate, construct galleries, and start laying eggs inside trees [61]. Therefore, the associates of bark beetle are not exposed to strong desiccation stress during departure from the vector. However, during the vector’s flight, the associates of bark beetle that stick to the backs of elytra are exposed to highly desiccating conditions.

With regard to phylogenetic relationships, the bark beetle associated *Bursaphelenchus* spp. are more basal than longhorn beetle associates (Fig 1). Thus, mobility and tolerance to desiccation provided by a well-developed basal striated zone in the associates of bark beetle is likely an ancestral (apomorphic) characteristic, serving as a kind of preadaptation for cerambycid host invasion.

The adult forms of *B*. *luxuriosa*e (and its tentative sister species, *B*. *doui*) were hypothesized to be “parasitic,” because they (1) were isolated from body cavity and (2) seemed active within the insect body, although obvious damage to insects was not confirmed [44, 45]. However, in the present study, the adult forms were mostly isolated from the tracheal systems and had a similar body cuticle structure as dauer juveniles, which was clearly different from the parasitic juveniles of *Parasitaphelenchus* sp. Therefore, although more detailed nutritional and physiological analyses are necessary, the insect-dependent adult forms of *B*. *luxuriosae* (and *B*. *doui*) may be phoretic rather than parasitic.

By contrast, the parasitic juveniles of *Parasitaphelenchus* sp. had no striated basal zone. This is probably because strong mobility and desiccation tolerance are not necessary for the parasitic form, settled in the hemocoel. However, if the parasitic (infective) juveniles enter the insect body as *Bursaphelenchus* dauers do, they must attach itself to the beetle body and enter the hemocoel. A possible explanation is that parasitic juveniles lose the basal striated zone after invasion. The juveniles show active movement during invasion, but after invasion, active movement and desiccation tolerance are unnecessary if they can avoid the host immune system, because they are assumed to float in the hemolymph. This fits well with our behavioral observations. *Bursaphelenchus* dauers are known to move actively after entering the vector body [62, 63], and they showed active movement immediately after insect dissection in the present study. It suggests that they may depart from their vectors at any time, which is difficult to predict. By contrast, the parasitic juveniles did not move immediately after host dissection, and not all individuals started moving within a day, suggesting that juveniles that moved into a vector’s intestine may need a certain period of “preparation time” to acquire mobility.

The structure of the nematode body cuticle is highly plastic [9] and is known to change before the stage of molting in many species. For example, the body cuticle of the first-stage juvenile of a human parasite filaria, *Wuchereria bancrofti*, loses the fiber zone found in early ontogenesis at the same stage [64], and infective juveniles of a root knot nematode, *Meloidogyne javanica*, lose the basal striation within a week of becoming a parasitic juvenile [51]. Similarly, second juveniles of a potato cyst nematode, *Globodera rostochiensis*, lose basal striation once the juveniles set up a feeding site [54]. Therefore, in the *Parasitaphelenchus* sp., it is quite probable that the basal striated zone is present before invasion and disappears after invasion.

The surface coat, the outermost layer of the epicuticle, is faintly stained and unclear in *Parasitaphelenchus* sp., while that of *Bursaphelenchus* spp. is relatively clear. This is likely because *Parasitaphelenchus* sp. has a chemically different surface coat from *Bursaphelenchus* spp., which streams away from the surface of the *Parasitaphelenchus* sp. during chemical fixation. Generally, in parasitic nematodes, the surface coat is involved in functions such as interacting with host immune defenses. For example, infective juveniles of the parasitic nematode *Toxocara canis* have mucins on their surface [65]. Because mucins are present on the luminal surface of host endothelial cells, it is possible that the surface coat disguises infective larvae, effectively evading the host immune system. A CuZn superoxide dismutase secreted by, and present on the surfaces of, adult male and female *Brugia pahangi* is presumed to neutralize superoxides generated by leukocytes, thereby contributing to parasite survival in its host by acting as an antioxidant factor [66]. Although further chemical and enzymatic studies are needed, the specific surface coat of *Parasitaphelenchus* sp. may similarly reflect adaptation to living in hemocoel.

*Parasitaphelenchus* spp. were reported to invade hosts as third-stage (infective) juveniles and molt to fourth-stage (parasitic) juveniles, enlarging considerably [28–30]. However, in this study, all *Parasitaphelenchus* sp. obtained from host beetles were the third-stage parasitic juveniles, and they molted to the fourth-stage mycophagous juveniles on fungal lawn. This result suggests that *Parasitaphelenchus* sp. have a different life cycle from other *Parasitaphelenchus* spp. Thus, it will be interesting to compare the body cuticle structures of third-stage (parasitic) juveniles of this *Parasitaphelenchus* sp. with third-stage (infective) and fourth-stage (parasitic) juveniles of other *Parasitaphelenchus* spp.

Phylogenetically, *Parasitaphelenchus* belong to clade 2 of *Bursaphelenchus* spp. (Fig 1). This means that the parasitism and cuticular structures of *Parasitaphelenchus* are believed to have derived from the fungal feeding/insect-dependent phoretic life history of *Bursaphelenchus* spp. Furthermore, several other *xylophilus* group species (clade 3) have parasitic juveniles [67, 68]. This suggests that insect parasitism has evolved independently at least twice in this subfamily. A detailed comparison among those parasitic juveniles will provide a better understanding of the convergence/divergence of the parasitic structures of parasitaphelenchids and other parasites. The drastic differences in cuticular structures found in the present study may represent nematode plasticity, one of the most important factors for their diversification.
